# Cell-based passive immunization for protection against SARS-CoV-2 infection

**DOI:** 10.1186/s13287-023-03556-5

**Published:** 2023-11-06

**Authors:** Evan Sawula, Shane Miersch, Eric D. Jong, Chengjin Li, Fang-Yu Chou, Jean Kit Tang, Reza Saberianfar, Jeffrey Harding, Sachdev S. Sidhu, Andras Nagy

**Affiliations:** 1 Institute of Medical Science, University of Toronto, Toronto, ON Canada; 2 Lunenfeld-Tanenbaum Research Institute, Sinai Health System, Toronto, ON Canada; 3 The Anvil Institute, University of Waterloo, Waterloo, ON Canada; 4 Department of Physiology, University of Toronto, Toronto, ON Canada; 5 Department of Obstetrics and Gynaecology, University of Toronto, Toronto, ON Canada; 6 Australian Regenerative Medicine Institute, Monash University, Melbourne, VIC Australia

**Keywords:** SARS-CoV-2, COVID-19, Neutralizing antibodies, Passive immunization, Cell therapy

## Abstract

**Background:**

Immunologically impaired individuals respond poorly to vaccines, highlighting the need for additional strategies to protect these vulnerable populations from COVID-19. While monoclonal antibodies (mAbs) have emerged as promising tools to manage infectious diseases, the transient lifespan of neutralizing mAbs in patients limits their ability to confer lasting, passive prophylaxis from SARS-CoV-2. Here, we attempted to solve this problem by combining cell and mAb engineering in a way that provides durable immune protection against viral infection using safe and universal cell therapy.

**Methods:**

Mouse embryonic stem cells equipped with our FailSafe™ and induced allogeneic cell tolerance technologies were engineered to express factors that potently neutralize SARS-CoV-2, which we call ‘neutralizing biologics’ (nBios). We subcutaneously transplanted the transgenic cells into mice and longitudinally assessed the ability of the cells to deliver nBios into circulation. To do so, we quantified plasma nBio concentrations and SARS-CoV-2 neutralizing activity over time in transplant recipients. Finally, using similar cell engineering strategies, we genetically modified FailSafe™ human-induced pluripotent stem cells to express SARS-CoV-2 nBios.

**Results:**

Transgenic mouse embryonic stem cells engineered for safety and allogeneic-acceptance can secrete functional and potent SARS-CoV-2 nBios. As a dormant, subcutaneous tissue, the transgenic cells and their differentiated derivatives long-term deliver a supply of protective nBio titers in vivo. Moving toward clinical relevance, we also show that human-induced pluripotent stem cells, similarly engineered for safety, can secrete highly potent nBios.

**Conclusions:**

Together, these findings show the promise and potential of using ‘off-the-shelf’ cell products that secrete neutralizing antibodies for sustained protective immunity against current and future viral pathogens of public health significance.

**Supplementary Information:**

The online version contains supplementary material available at 10.1186/s13287-023-03556-5.

## Background

Severe acute respiratory syndrome coronavirus 2 (SARS-CoV-2) entered humans in late 2019 and caused the ongoing coronavirus disease 2019 (COVID-19) pandemic. Although vaccines based on the ancestral SARS-CoV-2 spike protein lowered the risk of severe disease and death by early strains of the virus [1–4], their efficacy has been undermined by antigenically divergent viral variants that continue to emerge and spread globally [5–9]. Most recently, breakthrough infections caused by highly immune-evasive Omicron sublineages [10–14] have led to the emergency approval of Omicron-based mRNA booster vaccines. Further still, immunologically impaired individuals respond poorly to COVID-19 vaccines altogether and remain at risk of disease [15–20]. This underscores the need for new approaches to provide these vulnerable groups with immune protection.

Monoclonal antibodies (mAbs) are another promising countermeasure against viral pathogens. Many neutralizing mAbs to SARS-CoV-2 have been described to date [21–28], the most potent of which target the receptor-binding domain (RBD) of the spike protein and block its engagement with the angiotensin-converting enzyme 2 (ACE2) receptor to prevent viral entry. Like vaccines, several of these mAbs [29–33] have entered the clinic with unprecedented pace after being authorized for emergency use in numerous countries. While administering exogenous antibodies to populations that respond poorly to vaccines can confer pre-exposure prophylaxis against infection [31, 34], this passive immunity is limited by the lifespan of the mAbs in patients. Thus, there remains an unmet need to provide lasting protective immunity to these high-risk populations.

Cell-based therapies hold enormous promise in regenerative medicine and beyond, with several cell products already in clinical use and many others under clinical study for the treatment of a wide array of diseases [35–37] (NCT04802733, NCT04786262, and NCT01734733). Ongoing efforts to engineer safe and universal ‘off-the-shelf’ cells by us and others [38–43] point toward their increasing potential for widespread adoption in clinical settings. The ability to genetically modify cells to produce therapeutic factors is also an opportunity to develop cell products that can deliver biologics in vivo. Toward this goal, here we combined cell and antibody engineering to develop a cell-based passive immunization system to overcome the limited duration of protection afforded by passive mAbs.

We first show that mouse embryonic stem cells (mESCs) can be genetically engineered to secrete factors that potently neutralize SARS-CoV-2. We then show that long-lived transplants derived from these engineered cells can supply the factors in vivo at levels that could protect against SARS-CoV-2 infection. Furthermore, we observe high expression of the same neutralizing factors by engineered human-induced pluripotent stem cells (hiPSCs). Our work demonstrates the proof-of-principle that engineered human stem cells represent a source of therapeutic cells which have the potential to confer and maintain passive immunity as a pre-emptive measure against viral infection. To support our findings that high and immunologically sufficient antibody production does not necessarily require B cells, future work will explore optimal cell types for use in this system.

## Materials and methods

### Mouse ES cell culture

Genetically modified mESCs, derived from C57BL/6NCrl mice [44], were cultured on mitotically inactivated mouse embryonic fibroblasts in high-glucose DMEM (Gibco) with 15% fetal bovine serum (Gibco), leukemia-inhibiting factor produced at the Lunenfeld-Tanenbaum Research Institute (Sinai Health System, Toronto, Ontario), 2 mM glutamax (Gibco), 0.1 mM 2-mercaptoethanol (Gibco), 0.1 mM non-essential amino acids (Gibco), 1 mM sodium pyruvate (Gibco), and 50 µg/ml penicillin–streptomycin (Gibco) at 37 °C, 5% CO_2_. Culture medium was changed daily, and cells were passaged every 2–3 days with 0.25% trypsin–EDTA (Gibco) upon reaching 70–80% confluency.

To harvest the cell culture supernatant for in vitro characterization assays, 1 million mESCs were seeded in a 6-well culture dish. The culture medium was replaced 24 h post-seeding, and the supernatant was collected 48 h later.

### Construction of *piggyBac* transposon expression vectors

The SARS-CoV-2 neutralizing antibody 15033-7 was developed from a synthetic, phage-displayed antigen binding fragment (Fab) library in selections against the SARS-CoV-2 RBD, as previously described [23]. Using the DNA sequences encoding the variable heavy and light chain of 15033-7 and the fragment crystallizable (Fc) region of human IgG1, different single-chain antibody architectures were designed. Additionally, the coding sequences of the signal peptide and extracellular domain of the human ACE2 receptor (amino acids 1-740) were combined with the human IgG1 Fc region coding sequence.

Then, plasmids containing the full coding sequences of each nBio format in pDONR221 vectors were obtained by commercial gene synthesis (Twist Bioscience) and were separately cloned into *piggyBac* transposon destination vectors using the Gateway Cloning Kit (Thermo Fisher Scientific) as per the manufacturer’s protocol. Destination vectors comprised the constitutive CAG promoter driving transgene expression, which was transcriptionally linked to a downstream mCherry fluorescent reporter or a puromycin-resistance gene. Cloning of nBio sequences into expression vectors was verified by Sanger sequencing done at The Centre for Applied Genomics at the Hospital for Sick Children (Toronto, Ontario, Canada). Vectors were then transformed into DH5α competent *E. coli* (Invitrogen), and colonies were selected on LB agar plates containing ampicillin (Sigma). Ampicillin-resistant colonies were grown in LB broth and the plasmid DNA used for transfection into cells was then purified using the QIAprep Spin Miniprep Kit (Qiagen).

### Mouse ES cell transfection, selection, and cloning

Mouse ESCs in adherent 6-well culture dishes were separately transfected with 2 μg DNA (1.5 μg *piggyBac* expression vector containing sequence encoding each nBio and 0.5 μg episomal plasmid encoding hyperactive PBase (hyPBase) (The Sanger Center, pCMV-hyPBase) per manufacturer’s protocol using the JetPrime Transfection Kit (Polyplus Transfection). mESCs were transfected with expression vectors containing an mCherry fluorescent reporter, and transfected mESC pools were single-cell sorted by fluorescence-based cell sorting based on mCherry expression. Following sorting, several mCherry^high^ clones expressing each nBio format were expanded from 96-well culture dishes for in vitro characterization assays.

### Human iPS cell culture

Human iPSCs (NIH Regenerative Medicine Program, LiPSC-GR1.1), genetically modified by panCELLa Inc. (designated as PCA1-14) to contain the FailSafe™ system [42], were cultured on Geltrex (Thermo Fisher Scientific) in mTeSR1 medium (STEMCELL Technologies) with 50 μg/ml penicillin–streptomycin (Gibco) at 37 °C, 5% CO_2_. Culture medium was changed daily, and cells were passaged every 3–5 days with ReleSR (STEMCELL Tech.) upon reaching 70–80% confluency.

To harvest the cell culture supernatant for in vitro characterization assays, hiPSCs were dissociated to single cells using Accutase (STEMCELL Tech.) and 300,000 cells were seeded in hiPSC medium with 1 μM ROCK inhibitor (STEMCELL Tech.) in a 6-well culture dish. The culture medium was replaced 24 h post-seeding, and the supernatant was collected 48 h later.

### Human iPS cell transfection, selection, and cloning

Human iPSCs were separately transfected with 2.5 μg DNA (2 μg *piggyBac* expression vector containing sequence encoding several nBio formats and 0.5 μg hyPBase) per manufacturer’s protocol using the Lipofectamine 3000 Transfection Kit (Thermo Fisher). Transgene-containing hiPSCs were isolated by drug selection on transfected hiPSC pools for 7 days with puromycin (Gibco) at 1.5 μg/ml. Then, puromycin-resistant hiPSCs were plated at clonal densities (200 cells per 10 cm culture dish) and several clones were isolated and expanded for in vitro characterization.

### Mice

C57BL/6NCrl and NSG (NOD scid gamma/J#5557) mice (8–10 weeks old) were used for in vivo experiments. All animals were female, as sex was not considered a fundamental factor in this study. The mice were bred at the Toronto Centre for Phenogenomics (TCP; Sinai Health System) and housed at the TCP in a pathogen-free facility in micro-isolator cages (Techniplast) with individual ventilation at a maximum of 5 mice per cage on a 12-h light/dark cycle. All mouse procedures were performed in compliance with the Animals for Research Act of Ontario and the Guidelines of the Canadian Council on Animal Care and were approved by the TCP Animal Care Committee. All experiments performed were in adherence with the ARRIVE guidelines. Humane experimental endpoints were set at day 100 or day 150 after cell transplantation, or if teratomas surpassed 1600 mm^3^, upon which animals were anaesthetized under 4% isoflurane before being euthanized by cervical dislocation. The number of animals used in the study is indicated in the figure legends and was determined in accordance with similar studies in the field. No specific criteria were set for the inclusion and exclusion of animals, and all animals were included in subsequent analyses. Because of the nature of the experiments, blinding was not possible as the results were visible.

### Mouse ES cell transplantation assay

Matrigel Matrix High Concentration (Corning) was diluted 1:1 in cold DMEM and kept on ice. mESCs were dissociated, resuspended in mESC media, centrifuged, and washed twice in DMEM. Then, 5 million mESCs were diluted in 100 μl of the Matrigel-DMEM mixture and injected subcutaneously into the dorsal flank of the mice. Mice were anaesthetized during injections with isoflurane. Animals were randomly placed into control and treatment groups without specific methodology. Most teratomas formed 1–2-weeks following cell transplantation. Tissue size was measured using calipers and volume was calculated using the formula *V* = (*L* * *W* * *H*)*(π/6). All injected mESCs contained the FailSafe™ system (*HSV-TK* linked to *Cdk1* expression) [42] and thus teratomas were stabilized with ganciclovir (GCV) (Fresenius Kabi C315110) daily or every other day through intraperitoneal injections at 50 mg/kg in PBS. GCV treatment durations varied depending on teratoma volume and mouse strain and are indicated in the figures. Mouse plasma was obtained from peripheral blood, collected by the tail vein in 7.5% EDTA-coated tubes (Covidien), before cell transplantation and weekly thereafter.

### Bioluminescence imaging (BLI) of mice

For BLI, D-luciferin at 15 mg/ml (Xenolight D-Luciferin, PerkinElmer 122,799) in 100 μl PBS was injected intraperitoneally into the mice. Ten minutes following injection, mice were anaesthetized with isoflurane and imaged with the IVIS Lumina Imager (Perkin Elmer) at exposures between 30 s and 3 min. Binning was set to medium, and F/stop was set to 1. Images were acquired using the Living Images Software (Perkin Elmer). Mice were imaged weekly to every 2 weeks for up to 100- or 150-days following cell transplantation, depending on the experiment.

### Quantification of nBios by sandwich ELISA

An anti-human Fc fragment-specific capture antibody (not cross-reactive with mouse IgG) (Jackson Immunoresearch, 109-005-008) was immobilized in wells of a 384-well microplate from a 2 μg/ml solution in 1X PBS (pH 7.2) overnight at 4 °C. The antigen solution was removed, and the coated wells blocked by incubation with a solution of 5% skim milk in PBS for 1 h. Blocking solution was removed by 4X washing with 1X PBS 0.05% Tween (PBST) and serial twofold dilutions of either standard IgG, cell culture supernatant, or mouse plasma in PBST added to separate wells and incubated for 30 min to capture antibody. Wells were then washed 8X with PBST, and a 1:5000 solution of HRP-fused, anti-human Fc fragment-specific detection antibody (Jackson Immunoresearch, 109-035-008) was added and incubated for 30 min with shaking at room temperature. Plates were washed 8X with PBST, and a 1:1 solution of TMB substrate (KPL, KP-50-76-00) was added, allowing color to develop for 1 to 5 min before stopping the reaction with an equivalent volume of 1 M H_3_PO_4_ and reading the optical density at 450 nm.

### Production of pseudoviruses and luminescent infection assays

Pseudoviruses (virus-like particles pseudotyped with the SARS-CoV-2 spike protein) were prepared by co-transfection of HEK293 cells with 1 µg pNL4-3.luc.R-E- plasmid (luciferase expressing HIV-1 with defective envelope protein) (NIH AIDS Reagent Program, ARP2128) and 0.06 mg CMV promoter-driven plasmid encoding the spike protein using Lipofectamine 2000 transfection reagent (ThermoFisher, 11668027), exactly as described [23]. The infection assay was similarly performed as described [23], in brief, by pre-incubating pseudovirus with serial dilutions of nBio from either cell culture supernatant or mouse plasma in media at RT for 30 min, prior to addition to HEK293T cells stably expressing full-length human ACE2 protein. The cells and nBio/pseudovirus mixture were incubated at 37 °C with 5% CO_2_ for six hours, after which the media was replaced with fresh DMEM (10% FBS and 1% penicillin–streptomycin). After 72 h, DMEM was removed and DPBS (ThermoFisher) was added to cells before mixing with an equal volume of ONE-Glo EX Luciferase Assay System (Promega E8130), shaking for 5 min at room temperature, then reading the luciferase signal using a BioTek Synergy Neo plate reader (BioTek Instruments Inc.). The data were analyzed by GraphPad Prism Version 8.4.3 (GraphPad Software, LLC) to obtain IC_50_ and IC_90_ values.

### Pharmacokinetic modeling

To obtain an estimate of the number of cells required in a graft to achieve protective serum antibody titers, a one-compartment open model for continuous intravenous infusion [45] was applied, using the equation $C = \frac{{R_{0} }}{{{\text{Cl}}}}\left( {1 - e^{ - kt} } \right)$, where C = serum antibody concentration, $R_{0}$ = infusion rate (in other words, the rate of cellular secretion of the antibody), ${\text{Cl}}$ = clearance rate of serum antibody, $k$ = elimination rate constant ($\ln \left( 2 \right)$/$t_{1/2}$ of trastuzumab (28.5 days) [46]) and $t$ = time after cell transplantation. The rate of antibody secretion by transgenic FS-hiPSCs was determined by calculating the total amount of antibody secreted into the cell culture supernatant over the two-day period of conditioned media generation, relative to the number of cells present in the culture dish upon harvesting of the supernatant. Serum neutralizing antibody concentrations were then determined using this rate at multiple time points after cell transplantation, before which SARS-CoV-2 neutralizing titers were assumed to be zero. Protective serum antibody concentrations were based upon the expectation that a mAb’s IC_90_ concentration in lower respiratory sites will confer protection (Emergency Use Authorizations for all clinical-stage SARS-CoV-2 mAbs; www.fda.gov) and were back-calculated from previous modeling reports estimating that 6.5% of serum IgG antibodies penetrate respiratory sites [47, 48].

### Statistical analyses

All statistical analyses were carried out using GraphPad Prism Version 9.2 (GraphPad Software, LLC), and the specific tests performed are described in the corresponding figure legends.

## Results

### Transgenic mouse embryonic stem cells express SARS-CoV-2 neutralizing biologics

**Fig. 1:**
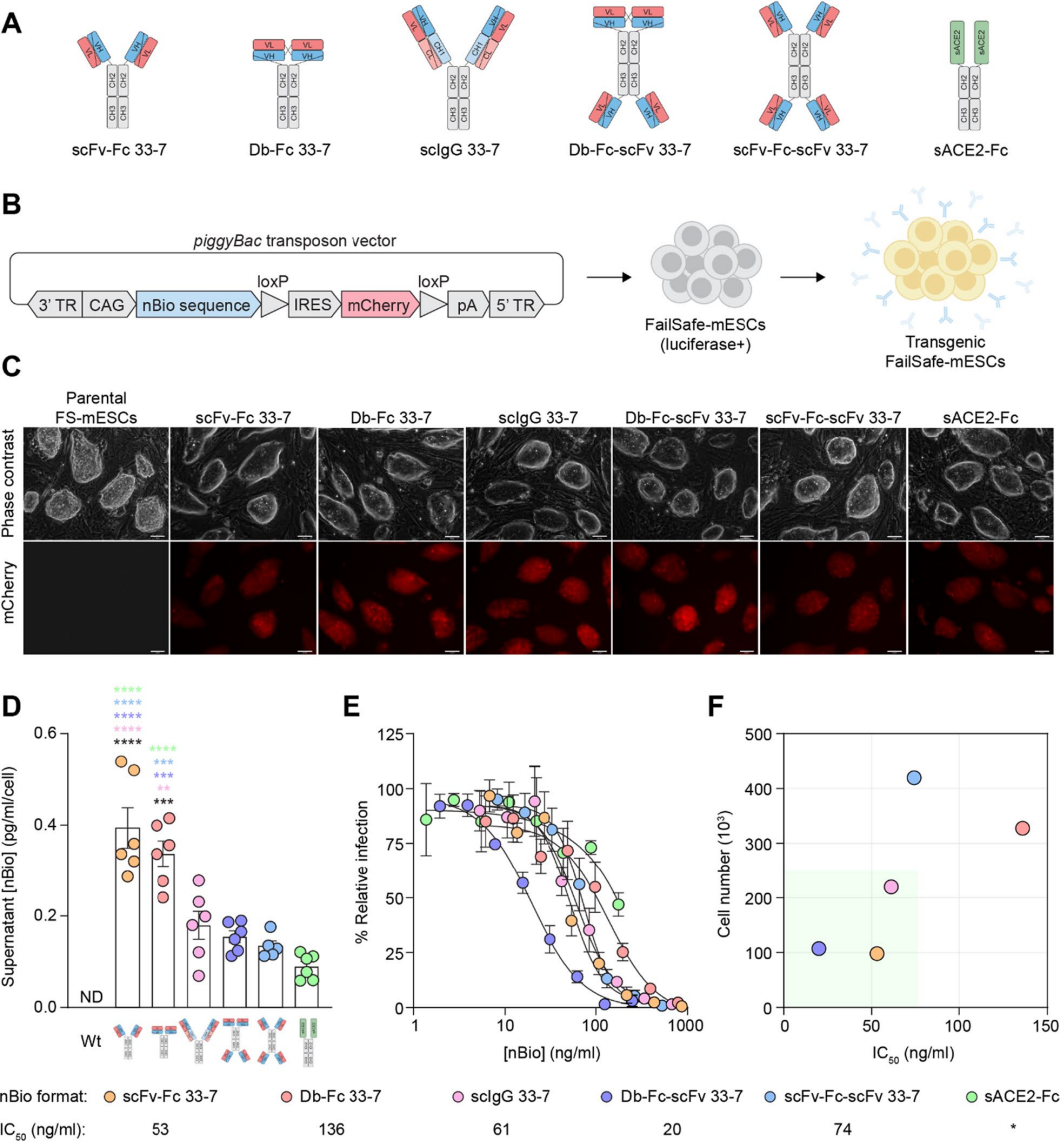
Development and in vitro characterization of transgenic mESCs. **A** Architectures of SARS-CoV-2 nBios encoded in human IgG1-based single-chain (sc) constructs. VL, variable light chain; VH, variable heavy chain; CL, constant light chain; CH, constant heavy chain. **B** The coding sequences of each SARS-CoV-2 nBio format were separately cloned into a *piggyBac* transposon expression vector. TR, terminal repeat; IRES, internal ribosome entry site; pA, polyadenylation. **C** Images of transgenic clonal mESCs expressing nBio transgenes linked to mCherry. All scale bars are 65 μm. **D** Quantification of nBios secreted into the culture supernatant by clonal transgenic mESCs by human anti-Fc ELISA. Each dot represents a separately generated clone expressing the same nBio format, according to its color. Bars represent the mean clonal secretion of nBio formats ± SEM of two independent experiments. The differences between nBio secretion between formats are not significant unless stated. Statistical significance was determined by one-way ANOVA with Tukey’s multiple comparisons test. **P = 0.0037; ***P < 0.0007; ****P < 0.0001. The black asterisks represent statistical significance relative to the Wt mESC control. Wt, wild-type; ND, not detected. **E** Neutralization of SARS-CoV-2 pseudovirus by mESC-derived nBios on human ACE2-overexpressing HEK293T target cells. Infection inhibition was measured as a function of nBio concentration in the supernatant of the highest expressing mESC clone of each format. Two independent experiments were performed with similar results. Curves were fit by nonlinear regression. Error bars represent SD. **F** Combined neutralization and secretion properties of each nBio format. The number of mESCs in culture required to reach half-maximal infection inhibition (IC_50_) by each nBio is plotted as a function of the IC_50_. The green box indicates the most ideal transgenic mESC line with respect to secretion and nBio potency. *An IC_50_ value for sACE2-Fc was not obtained due to incomplete neutralization in the assay at the concentrations tested

We designed alternative antibody formats by exploiting the modularity of antibody variable regions (Fig. 1A). Using a single human IgG1 framework, the variable regions of an RBD-directed human neutralizing antibody, 15033-7 (33-7) [23], were constructed into single-chain (sc) bivalent (scIgG, Db-Fc, scFv-Fc) and tetravalent (Db-Fc-scFv and scFv-Fc-scFv) formats. Synthetic constructs encoding each antibody format, as well as a soluble ACE2-Fc fusion protein (sACE2-Fc) [49, 50]—which together we call neutralizing biologics (nBios)—were individually cloned into *piggyBac* transposon vectors [51] in which nBio expression is driven by the constitutive CAG promoter [52] and transcriptionally linked to an mCherry fluorescent reporter by an IRES sequence (Fig. 1B).

To develop clonal mESC lines that secrete each nBio format, we used our FailSafe™ C57BL/6NCrl (B6) mESC (FS-mESC) line [42]. The FailSafe™ system links a negatively selectable (suicide) gene (herpes simplex virus type 1 thymidine kinase (HSV-TK)) to a cell-division essential gene (*Cdk1*, in this case), such that proliferating cells can be selectively eliminated with the HSV-TK pro-drug, ganciclovir (GCV). First, we introduced a luciferase transgene into these cells by *piggyBac* transposon-mediated transgenesis (Additional file 1: Fig. S1A). Then, we separately transfected the cells with each nBio vector and isolated transgenic clones with high nBio expression by FACS detection of high mCherry fluorescence (Fig. 1B and Additional file 1: Fig. S1B).

### SARS-CoV-2 nBios secreted by FS-mESCs are functional

Compared to the parental FS-mESC culture supernatant, in which, expectedly, we did not detect SARS-CoV-2 nBios, clonal transgenic FS-mESCs secreted nBios into the culture supernatant, with scFv-Fc and Db-Fc present at significantly (2- to 4-fold) higher levels on average compared to other formats (Fig. 1D). We then assessed the neutralizing activity of the culture supernatants of the highest-expressing clone of each nBio format. All formats neutralized ancestral SARS-CoV-2 in a lentivirus-based pseudovirus infection assay [23] (Fig. 1E). Half-maximal inhibitory concentration (IC_50_) values of the nBios ranged from 20 to 136 ng/ml and were comparable to the potencies of purified bivalent IgG 33-7 (IC_50_ = 83 ng/ml) and tetravalent 33-7 (IC_50_ = 10–40 ng/ml) previously determined against an authentic SARS-CoV-2 isolate (2019 nCoV/USA_WA1/2020) [23]. The Db-Fc-scFv format neutralized with the highest potency of all nBios expressed by transgenic FS-mESCs. In summary, safe mESCs can be genetically engineered to secrete functional and potent virus-neutralizing factors.

Given that each nBio format expressed by transgenic FS-mESCs possessed unique secretion and neutralization properties, we then determined which transgenic FS-mESC line to prioritize for in vivo studies by comparing the potency value of each nBio to its secretion value (Fig. 1F). In doing so, we aimed to establish which cell line had struck an ideal balance between these two properties, such that the most potent neutralization could be achieved with the least number of cells. Among nBios expressed by our clonal transgenic FS-mESCs, the scFv-Fc and Db-Fc-scFv formats had the best combination of cellular secretion and neutralization potency. On this basis, we selected these two formats for further in vivo investigation.

### Transgenic mESC transplants long-term supply functional SARS-CoV-2 neutralizing biologics in vivo

**Fig. 2:**
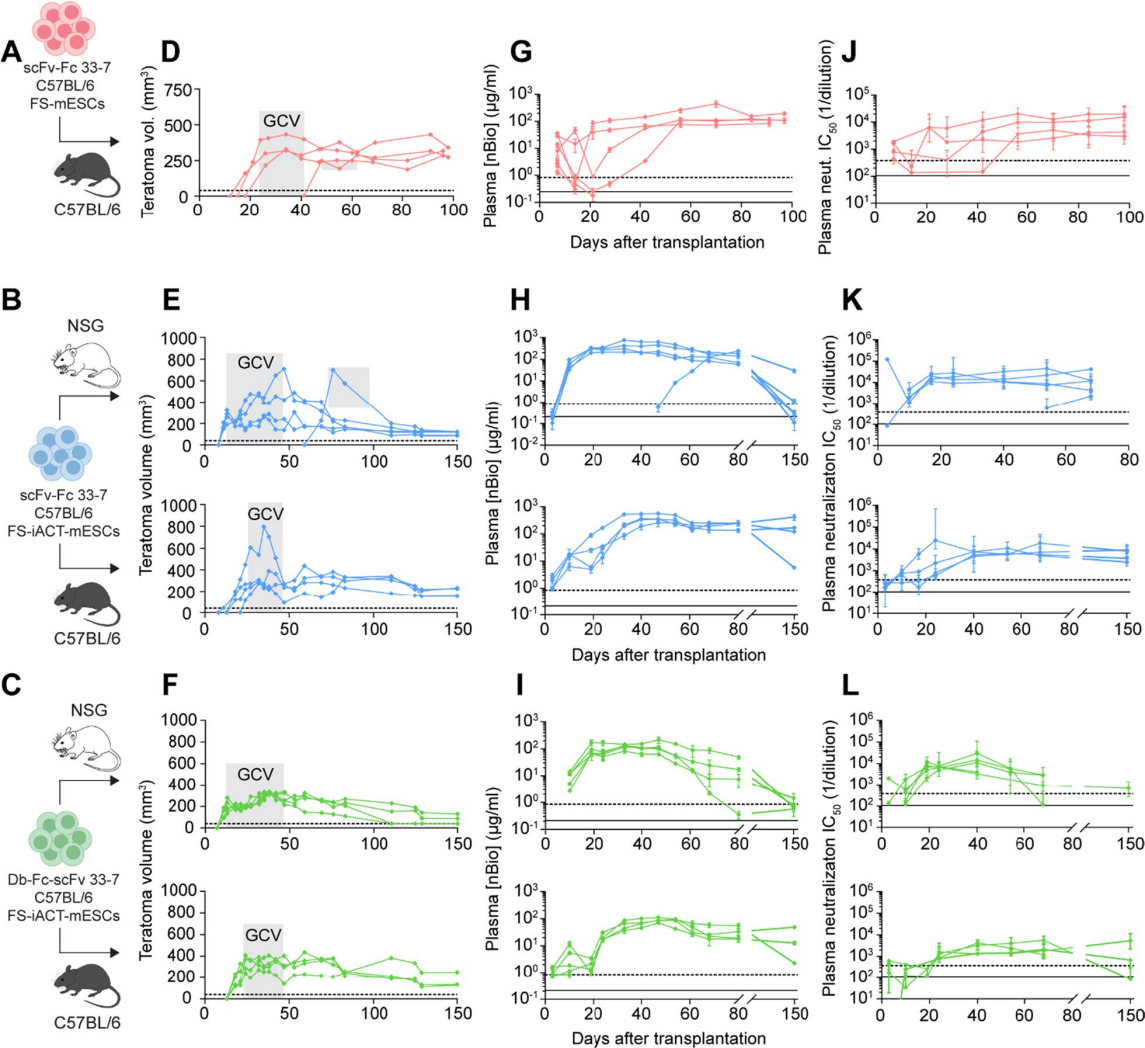
In vivo transplantation of transgenic mESCs. **A**–**C** Five million clonal transgenic B6 luciferase+ mESCs were subcutaneously injected into the dorsal flank of mice (**A**, scFv-Fc 33-7 FS-mESCs into B6 animals, *n* = 9; **B** and **C**, scFv-Fc and Db-Fc-scFv 33-7 FS-iACT-mESCs into NSG and B6 animals, *n* = 5 for each group). **D**–**F** Flank teratoma growth over the experimental period, measured by calipers. Ganciclovir (GCV) was administered to all mice over the indicated period to stabilize teratomas. Each line represents a single mouse per group. The dashed line indicates the minimum measurable teratoma volume with calipers, while teratomas are still present and palpable. **G**–**I** Levels of plasma nBios over time, assessed by anti-human Fc ELISA of the mouse plasma. The solid and dashed lines indicate previously defined minimum serum neutralizing antibody concentrations required to prevent weight loss (212 ng/ml) and reduce viral burden in the lung (851 ng/ml), respectively, in a mouse model of SARS-CoV-2 pathogenesis [56]. Error bars represent SD. **J**–**L** Neutralizing activity of mouse plasma over time against SARS-CoV-2 pseudovirus in vitro on human ACE2-overexpressing HEK293T target cells. Half-maximal neutralization (IC_50_) values were obtained by nonlinear regression and the neutralizing titer values were obtained by taking the reciprocal of the IC_50_ plasma dilution. The solid and dashed lines indicate previously defined minimum serum neutralizing titers required to prevent weight loss (104) and reduce viral burden in the lung (381), respectively, in a mouse model of SARS-CoV-2 pathogenesis [56]. Error bars represent 95% CI of neutralizing titers

Upon developing mESCs that express potent SARS-CoV-2 nBios and characterizing them in vitro, we next asked if the transgenic cells could supply protective titers of nBios in vivo. As a proof-of-concept approach, we first transplanted 5 million non-transgenic, parental FS-mESCs (Additional file 1: Figs. S2A and S4A, C), as well as 5 million clonal transgenic FS-mESCs expressing scFv-Fc 33-7 (Fig. 2A), into isogenic B6 recipients. Teratomas formed in 4 of 9 recipients of the transgenic FS-mESCs (Fig. 2D and Additional file 1: Fig. S2B) and we detected high levels of SARS-CoV-2 nBios in the plasma of these animals as early as 7 days after cell transplantation (the first time point assessed) (Fig. 2G). Given that teratomas comprise both quiescent and rapidly proliferating cell populations, a period of GCV administration enabled stabilization and control of teratoma size in all recipients by selective ablation of dividing cells, as we have done previously [42]. Plasma nBios reached a mean peak concentration of 195 µg/ml (range 88–452 µg/ml) and, because teratoma size was stabilized with GCV, persisted for at least 100 days (the length of the experiment) at stable levels between 84 and 198 µg/ml. Similarly, the plasma of these recipients demonstrated high neutralizing titers (reciprocal dilution for 50% neutralization of ancestral SARS-CoV-2 pseudovirus) over the same time period (Fig. 2J). Seven days after cell transplantation, a mean neutralizing titer of 1,180 (range 433–1900) was observed. The plasma maintained long-term neutralizing activity, displaying a mean titer of 10,500 (range 4300–19,700) at day 100. This was in contrast to recipients of non-transgenic, parental FS-mESCs, which, expectedly, in the lack of plasma nBios did not display SARS-CoV-2 neutralizing activity.

Although the efficiency of teratoma formation was lower than what would be expected in an isogenic setting, we were encouraged by those recipients in which successful teratoma growth and stabilization resulted in high and stable levels of plasma nBios over prolonged periods. Because the nBios are human proteins, it is possible that the cells producing these ‘xenogeneic’ factors were recognized as foreign and induced an immunogenic response that prevented the establishment of teratomas altogether in some recipients. To better protect the grafts from this immune response in such a scenario, we developed nBio-transgenic, FS-mESCs that also contain our induced allogeneic cell tolerance (iACT) technology (Klg-1 line in [40], denoted here as FS-iACT-mESCs) (Additional file 1: Fig. S3). Cells equipped with the iACT system express local-acting, immunomodulatory transgenes that promote the cells’ acceptance in allogeneic recipients without the need for host immune suppression.

We then transplanted 5 million parental FS-iACT-mESCs (Additional file 1: Figs. S2C and S4B) and 5 million clonal, nBio-transgenic FS-iACT-mESCs, separately expressing scFv-Fc 33-7 and Db-Fc-scFv 33-7, into isogenic B6 and, as a control, into immunodeficient NSG recipients (Fig. 2B, C). Transgenic FS-iACT-mESCs showed subcutaneous engraftment and teratoma growth (Fig. 2E, F and Additional file 1: Fig. S2D, E) with efficiency comparable to parental FS-iACT-mESCs (Additional file 1: Fig. S4D) in both immunodeficient and isogenic, immunocompetent hosts (growth in 4–5 out of 5 animals per group). GCV-stabilized teratomas persisted for 150 days (the length of the experiment) and circulating SARS-CoV-2 nBios were detected in the plasma as early as 3 days after transplantation (the first time point assessed) in most recipients at levels between 0.1 and 2.9 µg/ml, with the exception of all NSG recipients of Db-Fc-scFv transgenic mESCs, in which nBios were first detected 10 days post-transplantation (the next time point assessed after day 3) (Fig. 2H, I). Plasma nBio levels correlated well with teratoma volume and were also consistent with the in vitro secretion properties of the transplanted transgenic FS-iACT-mESC lines, where scFv-Fc was secreted at higher levels compared to Db-Fc-scFv. In vivo, scFv-Fc reached a mean peak plasma concentration of 401 µg/ml (range 241–767 µg/ml) between 3- and 10-weeks post-transplantation in NSG recipients and 380 µg/ml (range 259–559 µg/ml) between 6- and 8-weeks post-transplantation in B6 recipients. In comparison, Db-Fc-scFv reached a lower mean peak plasma concentration of 137 µg/ml (range 83 to 215 µg/ml) between 5- and 7-week post-transplantation in NSG recipients and 90 µg/ml (range 70–111 µg/ml) between 6- and 8-week post-transplantation in B6 recipients. Unsurprisingly, peak nBio titers correlated with the time periods when peak teratoma volumes were observed in transplant recipients. To contextualize the plasma concentrations reported here, peak serum antibody titers detected in a pharmacokinetic study in CD1 animals following intraperitoneal administration of a 2 mg/kg dose were around 30 µg/ml for both bi- and tetravalent analogs of 15,033 IgG (the parental clone of 15033-7) (data not shown). This suggests that therapeutically relevant titers are achieved and maintained following cell transplantation in mice.

By day 150, teratomas were stable at similar sizes in all mice (between 86 and 250 mm^3^). Volumes were slightly larger in B6 (mean 183 mm^3^) compared to NSG (mean 93 mm^3^) recipients. Consistent with this observation, the plasma of B6 recipients contained higher nBio concentrations (mean 178 µg/ml, range 5 to 415 µg/ml of scFv-Fc and mean 21 µg/ml, range 2–49 µg/ml of Db-Fc-scFv) compared to NSG recipients (mean 6.3 µg/ml, range 0.1–30 µg/ml of scFv-Fc and mean 0.84 µg/ml, range 0.5–1.5 µg/ml of Db-Fc-scFv).

Importantly, mouse plasma demonstrated long-term SARS-CoV-2 neutralizing activity over the 150-day period, which correlated well with nBio concentrations (Fig. 2K, L). Three days post-transplantation, the plasma containing scFv-Fc displayed modest neutralizing titers (84–275) in some NSG and all B6 recipients, and plasma containing Db-Fc-scFv displayed slightly higher neutralizing titers (177–2060). As nBios reached peak plasma concentrations, peak plasma neutralizing titers were also observed, confirming the cell-mediated production of functional antibodies. Neutralizing activity was higher in plasma containing scFv-Fc, reaching a mean titer of 31,300 (range 21,000–45,100) in NSG and 15,100 (range 6200–25,000) in B6 recipients. Although plasma-containing Db-Fc-scFv displayed less neutralizing activity in comparison, the observed peak titers were still high, reaching a mean of 14,000 (range 7300–29,600) in NSG and 4300 (range 1900–5700) in B6 recipients. Based on these observations, it appears that early after cell transplantation, when scFv-Fc and Db-Fc-scFv were present at similar concentrations in the plasma, neutralizing titers correlated with the neutralization potency of the nBio format itself given that plasma-containing Db-Fc-scFv displayed higher neutralizing activity at this time period. However, as peak nBio concentrations were reached in the plasma, the higher potency of Db-Fc-scFv does not appear to compensate for its lower production by cells, as scFv-Fc was present at higher concentrations and displayed higher neutralizing titers as a result.

At 150 days post-transplantation, the plasma retained neutralizing activity in all B6 recipients, displaying mean titers of 6500 (range 2500–9000) in the scFv-Fc group and 2000 (range 85–5300) in the Db-Fc-scFv group. However, the plasma of only one NSG recipient showed neutralization with a titer of 679. The plasma of the remaining NSG mice, which contained comparably lower nBio concentrations (0.1–1.5 µg/ml), failed to neutralize SARS-CoV-2 pseudovirus at day 150, likely because the plasma concentrations were not sufficient to prevent viral entry in our cell culture-based neutralization assay. Despite this, there may still be some degree of protection from disease by the low levels of circulating nBios upon viral challenge. Nonetheless, altogether, these results indicate that engineered cells are capable of sustainably supplying functional antibodies over long periods of time in vivo and that the iACT system promotes the long-term survival of the cells that supply these factors in mice.

### Development of transgenic human-induced pluripotent stem cells toward clinical relevance

**Fig. 3:**
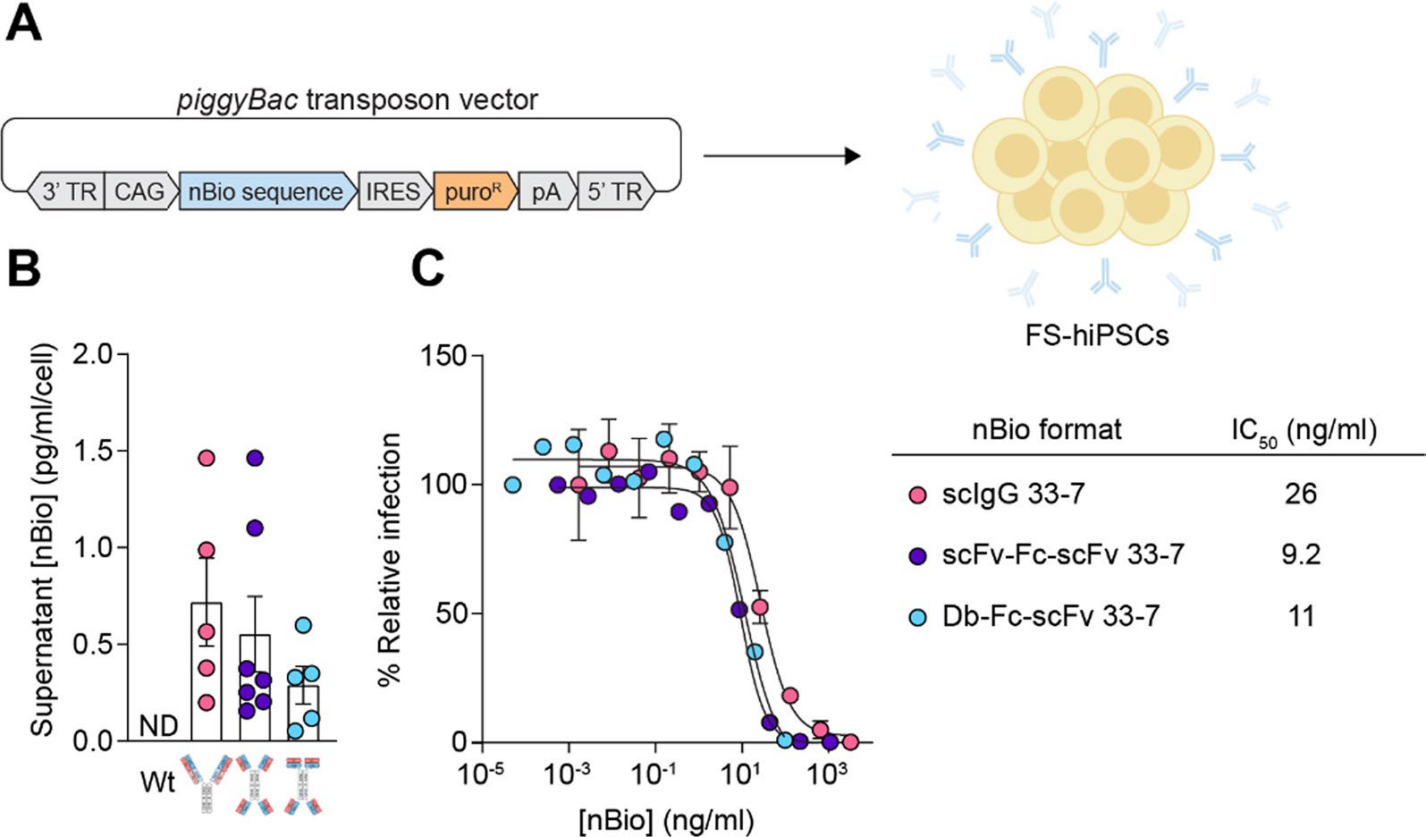
Development and in vitro characterization of transgenic hiPSCs. **A** The coding sequences of several of the described nBios were cloned into *piggyBac* transposon vectors in which nBio expression is driven by the constitutive CAG promoter and linked to a puromycin-resistance gene. Vectors were separately transfected into FS-hiPSCs and clonal cells expressing the nBio transgenes were isolated by puromycin selection. TR, terminal repeat; IRES, internal ribosome entry site; pA, polyadenylation. **B** Quantification of nBios secreted into the culture supernatant by clonal transgenic hiPSCs by anti-human Fc ELISA. Each dot represents a separately generated clone expressing the same nBio format, according to its color. Bars represent the mean clonal secretion of nBio formats ± SEM of two independent experiments. The differences between average secretion levels between nBio formats is not significant. Statistical significance was determined by one-way ANOVA with Tukey’s multiple comparisons test. Wt, wild-type; ND, not detected. **C** Neutralization of SARS-CoV-2 pseudovirus by hiPSC-derived nBios on hACE2-overexpressing HEK293T target cells. Inhibition of infection was measured as a function of nBio concentration in the supernatant of the highest expressing hiPSC clone of each format. Two independent experiments were performed with similar results. Curves were fit by nonlinear regression. Error bars represent SD

Finally, we aimed to build upon the results of our proof-of-concept studies using engineered mESCs and move toward clinical relevance by engineering human cells. To this end, we developed FailSafe™ human induced pluripotent stem cells (FS-hiPSCs) to express several of the SARS-CoV-2 nBio transgenes by *piggyBac* transposon-mediated transgenesis (Fig. 3A). Similar to the mouse system, following transfection and reporter selection, we evaluated several clonal transgenic FS-hiPSC culture supernatants for in vitro nBio secretion and neutralizing activity. SARS-CoV-2 nBios were present in the culture supernatants of the clones, with the bivalent scIgG 33-7 format secreted at the highest level on average compared to its tetravalent 33-7 counterpart formats, scFv-Fc-scFv and Db-Fc-scFv (Fig. 3B). Additionally, the supernatants of the highest-secreting clones of each format displayed potent neutralizing activity against SARS-CoV-2 pseudovirus, with IC_50_ values ranging from 9.2 to 26 ng/ml (Fig. 3C). Interestingly, nBios secreted by FS-hiPSCs displayed higher neutralization potencies compared to the same formats secreted by FS-mESCs, which may indicate that these human proteins are better functionally folded when secreted by human cells compared to mouse cells. Still, these results indicate that hiPSCs can also be engineered to secrete potent neutralizing biologics.

## Discussion

The urgent response by science, in concert with medicine and public health, to impact the course of the COVID-19 pandemic yielded countermeasures that were developed at extraordinary pace. Among these, mAbs arose not only as a disease treatment modality but also as valuable pre-exposure prophylactic agents [31, 53–55]. This offers hope to immunocompromised and other high-risk populations that do not share the same benefits afforded by COVID-19 vaccines as those with intact adaptive immune systems. Due to their limited lifespan in patients, the need for repeated mAb doses to achieve sustained protection represents a practical and economical hurdle that must be overcome for mAbs to realize their utility as widespread agents of long-term passive prophylaxis against viral infection and disease.

Here, we describe a strategy by which the temporary state of immunity afforded by passive mAbs can be overcome with long-lived cell transplants. Using a transposon-based gene delivery system, we show that mouse embryonic stem cells can be genetically modified to express various mAb formats that neutralize SARS-CoV-2 pseudovirus with high potency. We then demonstrate the feasibility of this approach in vivo—subcutaneously engrafted, transgenic stem cell-derived stable and safe tissues are capable of delivering functional biologics to mouse circulation at levels which far exceed that of previously defined minimum protective requirements for a mouse model of SARS-CoV-2 pathogenesis [56]. Moreover, these protective levels are maintained over extended periods of time. Given that neutralizing antibodies are considered a correlate of immune protection [57, 58], and protection from COVID-19 is attainable at low serum neutralizing titers [59], the plasma neutralizing activity we observed over several months here suggests that cells would be capable of conferring a pre-exposure prophylactic effect over long term. Notwithstanding, in vivo infection studies are needed to validate the protective efficacy of this approach. Finally, we pave a path toward clinical relevance by employing the same cell engineering methods to develop human stem cells that express potently neutralizing SARS-CoV-2 biologics. Altogether, this work also adds to the growing body of studies which use cells as delivery platforms for therapeutic factors. Others have enlisted this approach to overcome limitations associated with certain drugs, such as their bioavailability or capacity to localize to target tissues [60–63].

The objective of this study was to validate the concept of sustained, cell-mediated passive immunization by using pluripotent stem cell-derived tissues. Prior to this work, we have successfully engineered mouse and human pluripotent stem cells to reliably express transgenes [40] and have established methods to transplant these cells in ectopic sites and control their proliferation to achieve long-term tissue dormancy [40, 42]. We reasoned that employing these same strategies in this study would be an ideal way to investigate the ability of transgenic cells to deliver virus-neutralizing factors in vivo over long periods as a stable transplant. While we have demonstrated that transgenic mESCs and hiPSCs possess this capability, it is crucial to note the potential limitations of the direct use of pluripotent stem cells in clinical settings. While the FailSafe™ system effectively regulates the in vivo growth of tissue developing from pluripotent cells, these tissues’ cellular composition is somewhat random, which could pose repeatability issues and potential uncontrollable side effects. As we progress toward clinical application from this proof-of-principle study, the employment of safe, immune-evasive, in vitro generated, and well-defined cells that sustain longevity at the desired implantation site will be of paramount importance.

Using defined cell types with long-term survival capacity can mitigate cell death and ensure stable delivery of neutralizing antibodies from the tissue over long periods. In addition, an ideal cell type should also be well-connected to circulation, such that these two characteristics together can enable continuous protective immunity to be attained with a single transplantation. While long-lived plasma cells naturally represent an attractive candidate [64–66], our findings here also demonstrate the possibility of using a dormant, subcutaneous transplant to achieve such results. Both adipocytes and retinal pigment epithelium are extremely long-lived and quiescent cell types [67, 68] that, if capable of surviving in ectopic sites, could act as inert delivery vehicles for virus-neutralizing factors. In transplanting specific differentiated, non-proliferative cell types, peak neutralizing titers may also be reached sooner rather than relying on stem cell-derived tissues to first grow to sufficient sizes. This was observed in our study, where in some cases peak titers were observed several weeks after mESC transplantation. Although our FailSafe™ system provides a stringent layer of safety should some cells in the graft begin to proliferate and impend tumor development, the differentiated therapeutic cell types would also preclude the stabilization of the transplant with administration of GCV.

More generally, the possible risks and side effects associated with cell transplantation underscore the need for systems and technologies that ensure the safety of cellular medicines. For one, comprehensive safety assessments and pre-clinical studies—including rigorous testing for toxicity, immunogenicity, and other possible complications—should be conducted to evaluate the potential risks and adverse effects associated with engineered cells prior to transplantation. Transplanting cell types that are inherently less immunogenic can minimize the possibility of immune reactions toward the graft. Further, engineering immune-evasive properties into such cells can promote their acceptance in allogeneic hosts, yet these cells must also contain safety systems—such as our FailSafe™ system employed here—that offer control over undesired growth and proliferation upon transplantation, a process which may otherwise go unchecked if cells are able to evade recognition by the host’s immune system. Finally, patients must be closely monitored to detect and manage potential side effects as early as possible after transplantation. This may include follow-up tests and imaging at regular intervals to evaluate graft activity and any adverse events in vivo. Critically, all of these measures must be considered and implemented as the minimum prerequisites for cell-based therapies to become safe and universal, enabling them to enter clinical evaluation.

Also critical to this specific approach is achieving high antibody expression by our engineered cell products, such that protective titers can be reached with a clinically realistic number of cells in the graft. Many SARS-CoV-2 clinical mAbs have justified their dosing regimens on the basis that in vitro 90% inhibitory concentration (IC_90_) values in lower respiratory sites are likely to confer protection from COVID-19, even as pre-exposure prophylaxis in populations with compromised immune systems [47, 48, 69] (Emergency Use Authorizations for bamlanivimab and etesevimab, tixagevimab and cilgavimab, sotrovimab, and bebtelovimab; www.fda.gov). We applied these same estimates to our own in vitro results with FS-hiPSCs to approximate how many cells might be needed to achieve protective neutralizing antibody titers in a patient. Assuming a conservative expectation that 6.5% of serum mAbs penetrate respiratory sites [47, 48] and using the highest-expressing transgenic hiPSC clone of scFv-Fc-scFv 33–7 (IC_50_ = 9.2 ng/ml against ancestral SARS-CoV-2 pseudovirus), the minimum protective serum concentration is 586 ng/ml. By employing a model simulating continuous intravenous infusion (to reflect the constitutive secretion of antibody by a cell graft) [45] based on the pharmacokinetic properties of trastuzumab—from which the framework for mAb 33-7 is derived [23, 46]—we find that the minimum protective serum concentration can be reached with 250 million cells approximately 10 days after transplantation (Additional file 1: Fig. S5). Increasing the dose to 500 million, 750 million, or 1 billion cells reduces the time to minimum protective concentration to approximately 5, 4, or 3 days, respectively. These cell numbers are comparable to those used in current FDA-approved cell-based therapies (Approved Cellular and Gene Therapy Products; www.fda.gov) and the times to protection are reasonable in settings of pre-exposure prophylaxis. Of course, more nuanced modeling studies can substantiate the accuracy of these predictions, especially considering that the in vivo activity of an antibody and the cells secreting it may not precisely correlate with our observations in vitro. Together, this demonstrates in principle that it is possible to achieve protective antibody titers using a clinically realistic number of cells that express a potently neutralizing mAb.

Combining mAb and stem cell engineering strategies enabled us to develop stable cell lines which highly express SARS-CoV-2 neutralizing mAbs. Further exploration of these two engineering platforms can also address inherent limits to this type of approach. While the decline in nBio titers in some animals was likely in large part due to a decline in transplant size over time, silencing of *piggyBac* transgene integration sites may have also contributed to decreased nBio production by cell grafts. Teratomas are heterogeneous tissues comprising randomly differentiated cell types which possess diverse transcriptional landscapes. This phenomenon is also consistent with the fluctuations in nBio titers observed across animals with similar tissue sizes at various time points throughout the study. Alternatively, targeted transgene insertion into defined and active genomic sites using the CRISPR/Cas9 system, similar to strategies we have used previously [42], can ensure high, stable, and reliable antibody expression, especially if linked to a highly expressed, cell type-specific gene.

Finally, it is imperative to consider that an approach which uses a single antibody over long periods of time is especially threatened by both circulating and treatment-induced emergence of viral resistance. Selective immune pressure observed in chronic, immunocompromised patients undergoing mAb therapy has resulted in the emergence of escape mutants [70–72]. This, along with the considerable resistance to neutralizing antibodies elicited by vaccination or infection shown by emergent SARS-CoV-2 variants, emphasizes the importance of utilizing mAbs that maintain activity across diverse antigenic regions. Our engineered tetravalent antibodies display increased breadth and potency of neutralization [23] in a way that narrows the trade-off generally observed between these two properties.

Yet, because endogenous neutralizing antibodies to the spike protein, and particularly the RBD, are a driving force in the continued evolution of SARS-CoV-2, mAbs that display broad and potent activity across viral variants but bind such hotspot sites remain susceptible to escape. While combining separate RBD-directed antibodies into a single molecule using similar mAb design principles [73] represents a promising approach to withstand viral resistance, some of the broadest RBD-directed antibodies described to date that even recognize divergent sarbecoviruses [24, 25] are evaded by recent Omicron sublineages [11, 13, 14, 74]. Thus, additional strategies to further expand valency [75], target subdominant RBD epitopes [76] or other ‘cold spot’ regions of spike entirely—such as the highly conserved S2 stem helix [77, 78]—and more rigorously analyze the potential for escape may be needed to generate more resilient coverage across current and future viral variants. We envision that a detailed analysis of the epitopic landscape, coupled with rational antibody design strategies, will make it possible to engineer ‘escape-proof’ mAbs in such a way that their neutralization potency need not be sacrificed for their neutralization breadth. Further, although our sACE2-Fc construct displayed low expression and potency in our neutralization assays here, ACE2-based biologics represent a promising approach, insofar as they are capable of recognizing diverse SARS-CoV-2 variants given their universal need to bind the host ACE2 receptor [79, 80]. Though variants of ACE2 have been engineered to increase their affinity to spike (and thus their neutralization potency) [49], optimized residues may introduce new vulnerabilities to escape. Alternatively, host-directed antibodies targeting ACE2 have circumvented this limitation and shown in vivo utility [81, 82]. Overall, these types of biologics that are robust to viral escape and evolution represent promising candidates for this approach.

## Conclusions

Ultimately, in this study, we demonstrate the ability of safe and universal cells to deliver neutralizing antibodies over long periods of time in vivo, thereby having the potential to confer lasting passive prophylaxis. While this system is agnostic to the pathogen of interest and can be designed with effective antibodies to virtually any virus, its reach also extends beyond the realm of infectious disease. It represents a paradigm to treat any disease characterized by host factor deficiencies where cells can act as long-term in vivo delivery vehicles, including hemophilia, hypoparathyroidism, and beyond.

### Supplementary Information

**Additional file 1**. **Figure S1**: Isolation of clonal transgenic mESC pools. (A) Transfected FS-mESCs constitutively express luciferase from a *piggyBac* transposon vector for downstream in vivo tracking of cells. Enhanced firefly luciferase expression is linked to an eGFP fluorescent reporter within the expression cassette. TR, terminal repeat; IRES, internal ribosome entry site; pA, polyadenylation. (B) Flow cytometry plots for fluorescence-activated cell sorting of transfected FS-mESC pools. Gates indicate mESCs single-cell sorted for high mCherry and eGFP expression to establish clonal mESCs expressing each SARS-CoV-2 nBio format. The ‘wild-type’ (with respect to any neutralizing biologic transgene) and parental mESCs are mCherry^dim^ from the FailSafe^TM^ locus, in which mCherry is transcriptionally linked to the HSV-TK gene by a 2A peptide in a homozygous manner [42]. **Figure S2**: Bioluminescence image tracking of mESC transplant recipients. All transplanted mESCs constitutively expressed luciferase transgenes and were thus tracked over the experimental periods. Representative bioluminescence images of two animals per group are shown. (A) Parental FS-mESC and (B) clonal scFv-Fc 33-7 transgenic FS-mESC lines transplanted into B6 recipients (n = 8 and n = 9 animals, respectively). (C) Parental FS-iACT-mESC line and (D and E) clonal transgenic FS-iACT-mESC lines expressing scFv-Fc and Db-Fc-scFv 33-7, respectively, transplanted into NSG and B6 recipients (all n = 5 animals per group). Luc, luciferase. **Figure S3**: Development of clonal transgenic FS-iACT mESCs. (A) A B6-derived FS-iACT-mESC line (luciferase+, upper expression vector) was separately transfected with the piggyBac transposon expression vectors containing scFv-Fc 33-7 and Db-Fc-scFv 33-7 (lower vector, nBio expression linked to an mCherry fluorescent reporter). (B) Flow cytometry plots for fluorescence-activated cell sorting of the two transfected FS-mESC pools. Upper right gates indicate mESCs single-cell sorted for high mCherry and eGFP expression to establish clonal mESCs expressing the two nBio formats. (C) Images of transgenic clonal mESCs expressing the two different nBio transgenes linked to mCherry. All scale bars are 65 μm. Wt, wild-type. (D) Quantification of scFv-Fc and Db-Fc-scFv 33-7 secreted into the culture supernatant by clonal transgenic FS-iACT-mESCs by anti-human Fc ELISA. Each dot represents a separately generated clone expressing the same nBio format, according to its color. Bars represent the mean clonal secretion of nBio formats ± SEM. Statistical significance was determined by one-way ANOVA test with Tukey’s multiple comparisons test. **P = 0.005; ***P < 0.0001. ns, not significant; ND, not detected. **Figure S4**: In vivo transplantation of unmodified, parental mESCs. (A-B) Five million parental B6 luciferase+ mESCs were subcutaneously injected into the dorsal flank of mice (A, FS-mESCs into B6 animals, n = 8; B, FS-iACT-mESCs into NSG and B6 animals, n = 5 for each group). (C-D) Flank teratoma growth over the experimental period, measured by calipers. Ganciclovir (GCV) was administered to all mice over the indicated period to stabilize teratomas. Each line represents a single mouse per group. The dashed line indicates the minimum measurable teratoma volume with calipers, while teratomas are still present and palpable. As expected, no SARS-CoV-2 nBios were detected in the plasma of these mice by anti-human Fc ELISA.

## Data Availability

The datasets used and analyzed during the current study are available from the corresponding author upon reasonable request.

## Abbreviations

ACE2: Angiotensin-converting enzyme 2
B6: C57BL/6NCrl
COVID-19: Coronavirus disease 2019
Db: Diabody
FS: FailSafe™
GCV: Ganciclovir
hiPSC: Human-induced pluripotent stem cell
iACT: Induced allogeneic cell tolerance
IC_50_: Half-maximal inhibitory concentration
IC_90_: 90% Inhibitory concentration
mAb: Monoclonal antibody
mESC: Mouse embryonic stem cell
nBio: Neutralizing biologic
NSG: NOD scid gamma
RBD: Receptor-binding domain
SARS-CoV-2: Severe acute respiratory syndrome coronavirus 2
sc: Single-chain
HSV-TK: Herpes simplex virus type 1 thymidine kinase
