# Supplementary material for: Cell-based passive immunization for protection against SARS-CoV-2 infection
Source: Stem Cell Res Ther. 2023 Nov 6;14:318. doi: 10.1186/s13287-023-03556-5 (PMC10629160; doi:10.1186/s13287-023-03556-5)
Supplement: Supplementary file 1 — Additional file 1. Figure S1: Isolation of clonal transgenic mESC pools. (A) Transfected FS-mESCs constitutively express luciferase from a piggyBac transposon vector for downstream in vivo tracking of cells. Enhanced firefly luciferase expression is linked to an eGFP fluorescent reporter within the expression cassette. TR, terminal repeat; IRES, internal ribosome entry site; pA, polyadenylation. (B) Flow cytometry plots for fluorescence-activated cell sorting of transfected FS-mESC pools. Gates indicate mESCs single-cell sorted for high mCherry and eGFP expression to establish clonal mESCs expressing each SARS-CoV-2 nBio format. The ‘wild-type’ (with respect to any neutralizing biologic transgene) and parental mESCs are mCherrydim from the FailSafeTM locus, in which mCherry is transcriptionally linked to the HSV-TK gene by a 2A peptide in a homozygous manner [42]. Figure S2: Bioluminescence image tracking of mESC transplant recipients. All transplanted mESCs constitutively expressed luciferase transgenes and were thus tracked over the experimental periods. Representative bioluminescence images of two animals per group are shown. (A) Parental FS-mESC and (B) clonal scFv-Fc 33-7 transgenic FS-mESC lines transplanted into B6 recipients (n = 8 and n = 9 animals, respectively). (C) Parental FS-iACT-mESC line and (D and E) clonal transgenic FS-iACT-mESC lines expressing scFv-Fc and Db-Fc-scFv 33-7, respectively, transplanted into NSG and B6 recipients (all n = 5 animals per group). Luc, luciferase. Figure S3: Development of clonal transgenic FS-iACT mESCs. (A) A B6-derived FS-iACT-mESC line (luciferase+, upper expression vector) was separately transfected with the piggyBac transposon expression vectors containing scFv-Fc 33-7 and Db-Fc-scFv 33-7 (lower vector, nBio expression linked to an mCherry fluorescent reporter). (B) Flow cytometry plots for fluorescence-activated cell sorting of the two transfected FS-mESC pools. Upper right gates indicate mESCs single-cell sorted [file 13287_2023_3556_MOESM1_ESM.docx]

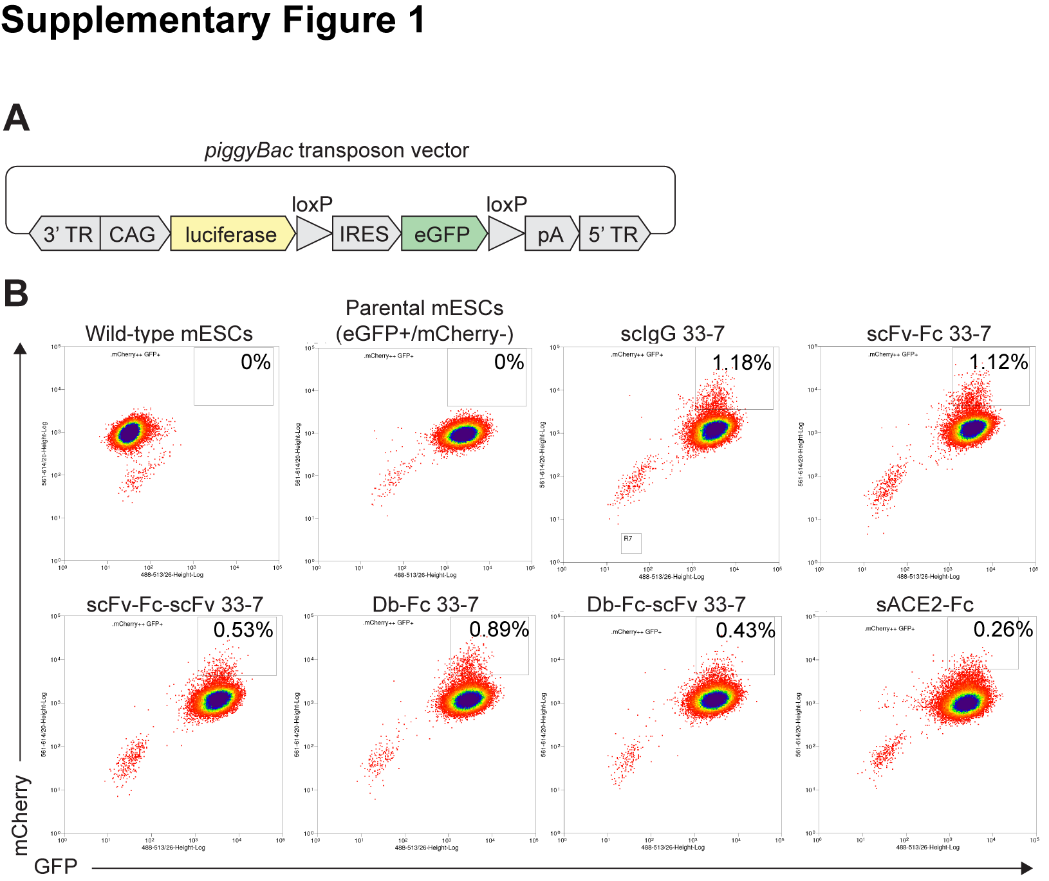


**Supplementary Figure 1. Isolation of clonal transgenic mESC pools. (A)** Transfected FS-mESCs constitutively express luciferase from a *piggyBac* transposon vector for downstream *in vivo* tracking of cells. Enhanced firefly luciferase expression is linked to an eGFP fluorescent reporter within the expression cassette. TR, terminal repeat; IRES, internal ribosome entry site; pA, polyadenylation. **(B)** Flow cytometry plots for fluorescence-activated cell sorting of transfected FS-mESC pools. Gates indicate mESCs single-cell sorted for high mCherry and eGFP expression to establish clonal mESCs expressing each SARS-CoV-2 nBio format. The ‘wild-type’ (with respect to any neutralizing biologic transgene) and parental mESCs are mCherry^dim^ from the FailSafe^TM^ locus, in which mCherry is transcriptionally linked to the HSV-TK gene by a 2A peptide in a homozygous manner [42].


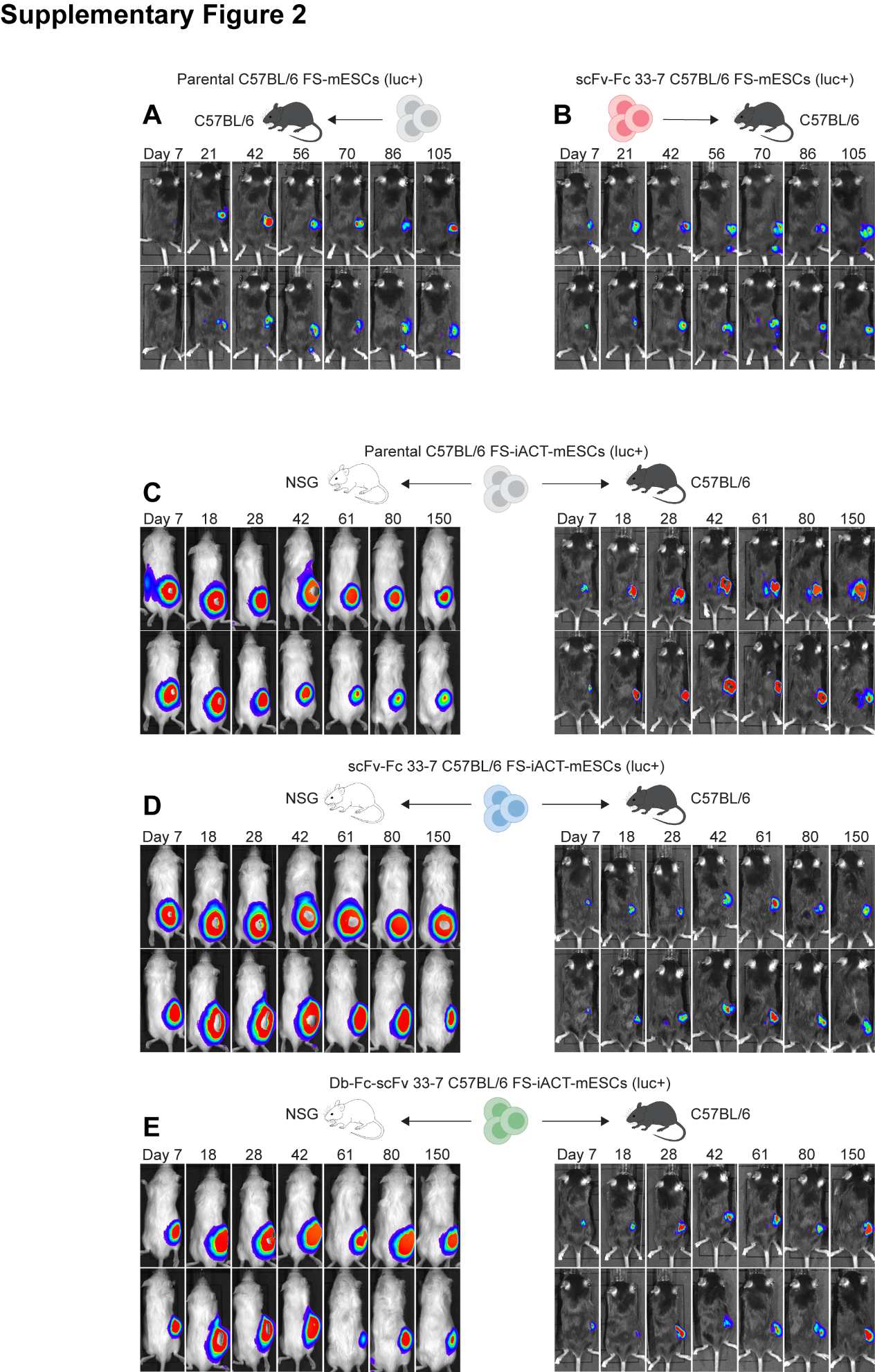


**Supplementary Figure 2. Bioluminescence image tracking of mESC transplant recipients.** All transplanted mESCs constitutively expressed luciferase transgenes and were thus tracked over the experimental periods. Representative bioluminescence images of two animals per group are shown. **(A)** Parental FS-mESC and **(B)** clonal scFv-Fc 33-7 transgenic FS-mESC lines transplanted into B6 recipients (*n* = 8 and *n* = 9 animals, respectively). **(C)** Parental FS-iACT-mESC line and **(D and E)** clonal transgenic FS-iACT-mESC lines expressing scFv-Fc and Db-Fc-scFv 33-7, respectively, transplanted into NSG and B6 recipients (all *n* = 5 animals per group). Luc, luciferase.

**
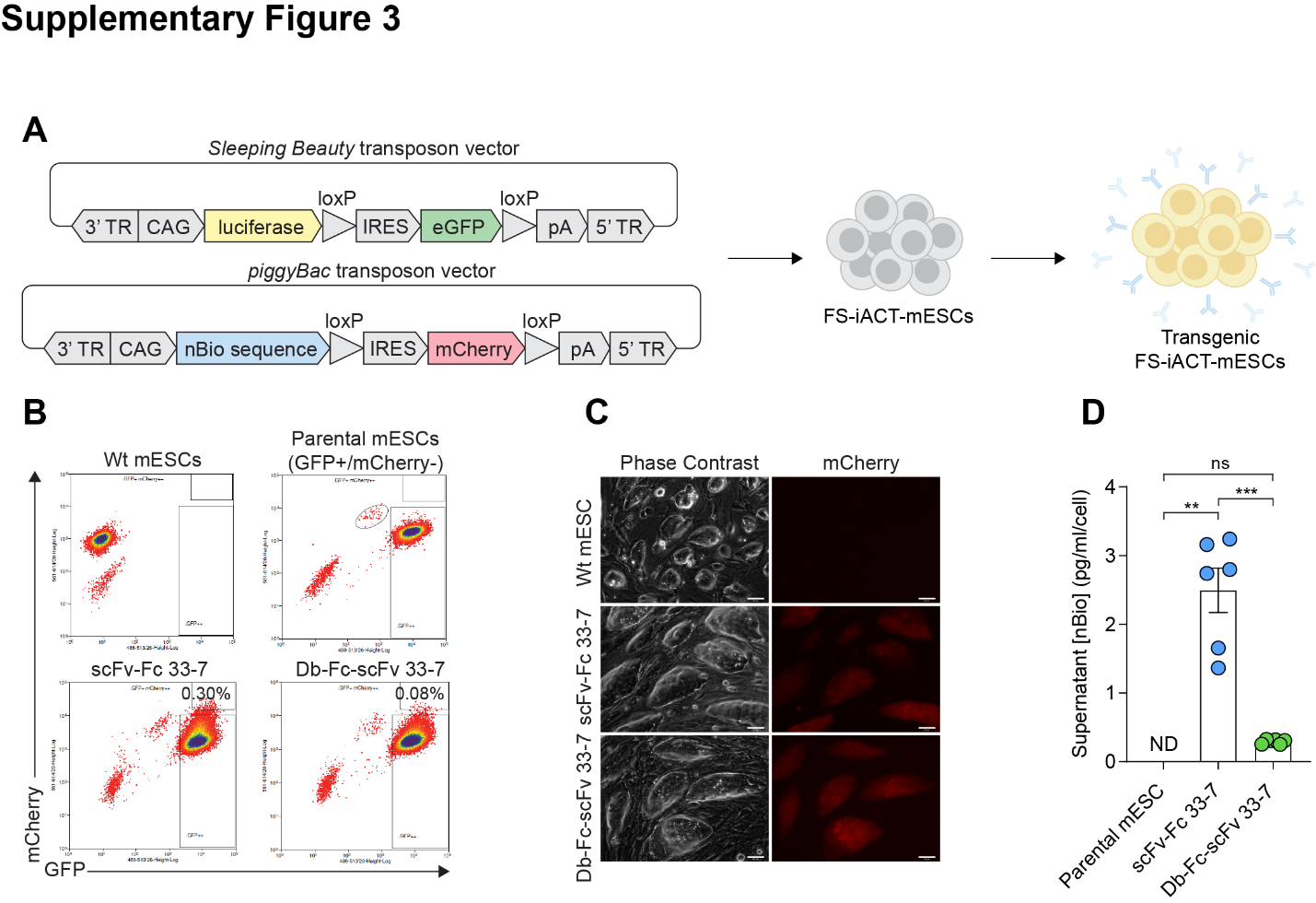
**

**Supplementary Figure 3. Development of clonal transgenic FS-iACT mESCs. (A)** A B6-derived FS-iACT-mESC line (luciferase+, upper expression vector) was separately transfected with the *piggyBac* transposon expression vectors containing scFv-Fc 33-7 and Db-Fc-scFv 33-7 (lower vector, nBio expression linked to an mCherry fluorescent reporter). **(B)** Flow cytometry plots for fluorescence-activated cell sorting of the two transfected FS-mESC pools. Upper right gates indicate mESCs single-cell sorted for high mCherry and eGFP expression to establish clonal mESCs expressing the two nBio formats. **(C)** Images of transgenic clonal mESCs expressing the two different nBio transgenes linked to mCherry. All scale bars are 65 μm. Wt, wild-type. **(D)** Quantification of scFv-Fc and Db-Fc-scFv 33-7 secreted into the culture supernatant by clonal transgenic FS-iACT-mESCs by anti-human Fc ELISA. Each dot represents a separately generated clone expressing the same nBio format, according to its colour. Bars represent the mean clonal secretion of nBio formats ± SEM. Statistical significance was determined by one-way ANOVA test with Tukey’s multiple comparisons test. **P = 0.005; ***P < 0.0001. ns, not significant; ND, not detected.


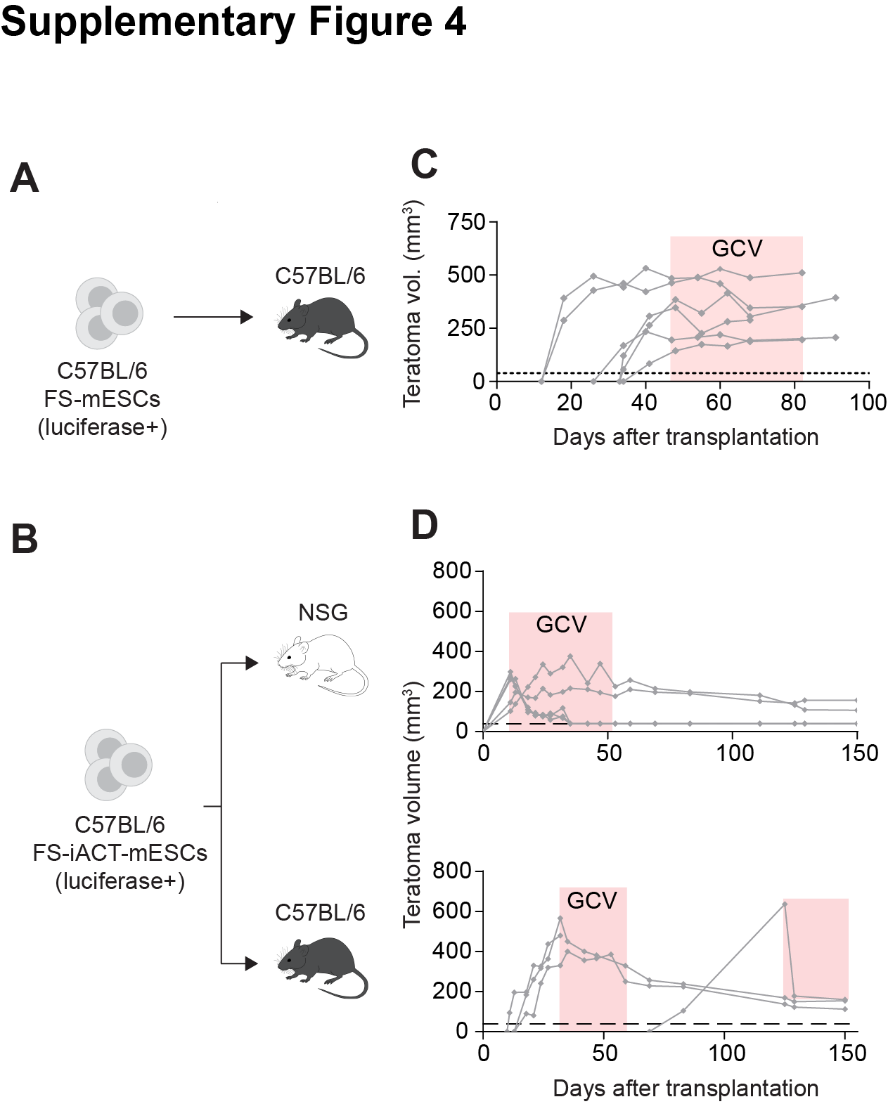


**Supplementary Figure 4. *In vivo* transplantation of unmodified, parental mESCs. (A-B)** Five million parental B6 luciferase+ mESCs were subcutaneously injected into the dorsal flank of mice (**A**, FS-mESCs into B6 animals, *n* = 8; **B**, FS-iACT-mESCs into NSG and B6 animals, *n* = 5 for each group). **(C-D)** Flank teratoma growth over the experimental period, measured by calipers. Ganciclovir (GCV) was administered to all mice over the indicated period to stabilize teratomas. Each line represents a single mouse per group. The dashed line indicates the minimum measurable teratoma volume with calipers while teratomas are still present and palpable. As expected, no SARS-CoV-2 nBios were detected in the plasma of these mice by anti-human Fc ELISA.


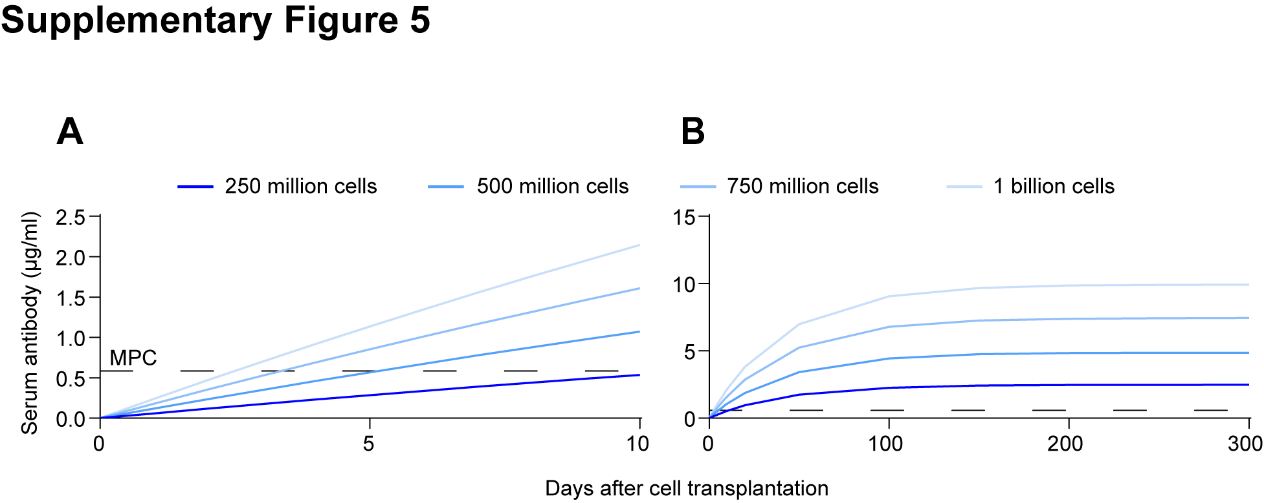


**Supplementary Figure 5.** **Estimating the number of grafted cells required for protection.** Serum neutralizing antibody concentrations over a 10 **(A)** and 300 **(B)** day period after transplanting transgenic FS-hiPSCs were calculated using a one-compartment pharmacokinetic model based on continuous intravenous infusion to mimic the constitutive secretion of antibody by the cell graft. The rate of antibody secretion by cells was determined by *in vitro* characterization studies of the transgenic FS-hiPSCs. The number of grafted cells tested in the model fall within the range of cell therapy products currently in clinical use. MPC, minimum protective concentration of antibody in serum, calculated based on the expectations that an IC_90_ concentration (of scFv-Fc-scFv 33-7, in this case) in respiratory sites will confer protection and 6.5% of serum mAbs will penetrate these sites [47,48].
